# The non-local effects of 7-week foot sole static stretching and foam rolling training on shoulder extension range of motion

**DOI:** 10.3389/fspor.2023.1335872

**Published:** 2024-01-12

**Authors:** Andreas Konrad, Marina Reiner, Josefina Manieu, Josef Fischer, Adrian Schöpflin, Markus Tilp, David G. Behm

**Affiliations:** ^1^ Institute of Human Movement Science, Sports and Health, University of Graz, Graz, Austria; ^2^ School of Human Kinetics and Recreation, Memorial University of Newfoundland, St. John’s, NL, Canada

**Keywords:** stretch training, roller massage, flexibility, crossover, global effects

## Abstract

Static stretching and foam rolling can increase the range of motion (ROM) of a joint acutely as well as chronically. Although studies have reported ROM increases of a non-stretched heterologous muscle (non-local) following an acute static stretching or foam rolling session, these effects have not been studied for long-term training interventions. Therefore, the purpose of this study was to investigate the effects of a comprehensive 7-week static stretching and foam rolling training program of the foot sole on shoulder extension ROM. A total of 33 healthy, physically active participants (20 male) were assigned to either the intervention (*n* = 19) or control (*n* = 14) group. The intervention group performed a 7-week combined static stretching and foam rolling intervention comprising three sessions a week, including three exercises of the foot sole for 5 min each. Before and after the intervention period, the shoulder extension ROM was tested with three-dimensional (3D) motion caption. The level of significance for all statistical analyses was set to *ρ *≤ 0.05. There was no significant time (*p* = 0.70, F1, 31 = 0.157; η2 = 0.005) or time×group effect (*p* = 0.38, F1, 31 = 0.785; *η*^2^ = 0.025) in shoulder extension ROM, indicating no ROM changes in the intervention or the control group. Although previous studies on the acute effects of stretching and foam rolling reported non-local increases in ROM in heterologous muscles, this study could show that such effects do not occur after chronic SS and foam rolling training for 7 weeks. Consequently, if the goal is to chronically increase the ROM of a specific joint, it is recommended to directly treat the muscles of interest.

## 1 Introduction

Static stretching is the most common stretching technique and is able to increase the range of motion (ROM) of a joint acutely following a single bout of stretching (1–3) as well as chronically following stretch training for several weeks (4–7). In recent years, foam rolling has attracted attention in sports practice as well as in research and it has been seen as a potential substitute/alternative for static stretching. Indeed, recent meta-analyses reported that compared with static stretching, foam rolling showed a similar acute effect on ROM (8, 9). Furthermore, a combination of both strategies also leads to an acute increase in ROM (10). If only foam rolling is applied frequently for several weeks (chronic), a recent meta-analysis showed an increase in joint ROM (11).

Besides these local effects of stretching and foam rolling on ROM, a single static stretching exercise as well as foam rolling exercise not only induces a change in ROM of the targeted joint but also impacts other non-adjacent joints (i.e., separate joints, not neighboring), leading to increased flexibility (12–14). A potential mechanism is an increased global stretch (pain) tolerance or even just warm-up effects (i.e., increase in body temperature) following both applications, which allows a higher ROM at locations in the body that did not receive any direct stretch or foam rolling stimulus (12, 15).

Considering static stretch and foam rolling training for several weeks, some studies reported contralateral (i.e., cross-over; cross-education) effects [static stretching (16–18), foam rolling (19, 20)]. Consequently, these studies indicated that with unilateral stretching or foam rolling of a muscle for several weeks, the contralateral homologous muscle (i.e., the same muscle on the other side of the body) may also increase ROM. These contralateral effects were again mainly attributed to altered pain perception (17, 19).

Recently, a study investigated a 7-week combined stretch and foam rolling training of the foot sole for 3 × 15 min a week on dorsiflexion ROM (21). The authors hypothesized that a remote effect (i.e., effect along a myofascial chain) along the myofascial superficial backline might occur. The superficial backline is purported to be composed of myofascial chains linked via connective tissues (22, 23). However, results revealed only a tendency (*p* = 0.08) of a statistical interaction effect for ankle dorsiflexion ROM following the intervention. Although, when considering the pre-to-post comparison, only an increase in ankle ROM in the intervention group of 2.0° (*p* = 0.05; *d* = 0.5) was detected, without a change in the control group (−0.5°, *p* = 0.62; *d* = 0.1) (21).

While a chronic effect following static stretching (16–18) and foam rolling (19) on the ROM in contralateral homologous muscles has been shown, to date, there is no such evidence in heterologous (i.e., different muscles indifferent body region) regions following a combined treatment of static stretching and foam rolling (e.g., stretching and foam rolling treatment of the lower body and testing for ROM changes in the upper body). A potential change in ROM in heterologous muscles following such treatments could be of great importance for immobilized areas of the body such as casts or wheelchairs.

Therefore, the purpose of this study was to investigate the effects of a comprehensive 7-week combined static stretching and foam rolling training of the foot sole (three sessions/week for 15 min/session) on shoulder extension ROM. We hypothesized that the intervention on the foot sole would not induce significant changes in shoulder extension ROM.

## 2 Methods

### 2.1 Experimental design

Each participant visited the laboratory three times: a familiarization session, a presession before the intervention, and a postsession after the intervention. In the presession, the participants were assigned to either the intervention (*n* = 19) or the control group (*n* = 14). Please note that only the participants of the intervention group were part of another, bigger project where some results have been previously published (21). At the beginning of the sessions, each participant did a 4-min warm-up procedure of synchronous arm rotations with extended elbow joints and the greatest radius possible. The arm rotations were performed for 2 min in each direction, alternating directions every minute, at a speed of 120°/s (= 20 rotations per minute). The movements were standardized via auditive metronome signals. The outcome measure was shoulder extension ROM tested on the dominant arm (i.e., used for writing).

### 2.2 Participants

Although no similar study approach exists, in a previous study, a significant contralateral effect of the dorsiflexion ankle ROM was seen following a static stretch training program on the triceps surae muscle with a large effect size (Cohen's *d* = 1.58) (18). Using the conversion of (24), we get from a Cohen's *d* of 1.58 to an *f*-value for repeated measures analysis of variance (ANOVA)of 0.79. Hence, we estimate a minimum sample size of 12 participants (= six for each group) for this study [repeated measures (between factors) ANOVA (two groups × two measures), *f* = 0.79, *α* = 0.05, 1−*β* = 0.8, correlation among repeated measures = 0.5] using G*Power software (25). However, to be safe and to account for possible drop-outs, 33 healthy physically active participants (female: *n* = 13, age: 27.7 ± 4.2 years, weight: 62.0 ± 9.2 kg, height: 165.6 ± 6.3 cm; male: *n* = 20, age: 26.5 ± 4.4 years, weight: 77.7 ± 10.6 kg, height: 177.8 ± 6.1 cm) volunteered for this study. A comparison between the participants in the intervention group and the control group is provided in Table 1. None of the participants had any injury of the upper or lower extremities in the 6 months prior to the study. Participants were not informed about the hypothesis of the project. A written informed consent form was signed by the participants. The ethical approval was given by the ethical commission of the University of Graz (approval code GZ. 39/68/63 ex 2020/2021), and the protocol was chosen to meet the requirements of the Declaration of Helsinki.

**Table 1:** Comparison between the baseline characteristics of the intervention group and the control group.

	Intervention group	Control group	*P*-value
Age (years)	27.13 ± 4.66	26.47 ± 3.90	0.67
Weight (kg)	71.21 ± 9.64	71.89 ± 16.20	0.88
Height (m)	174.05 ± 6.88	171.57 ± 10.57	0.42
Male/female	11/8	9/5	

### 2.3 Procedures

#### 2.3.1 Shoulder extension ROM

**Figure 1:**
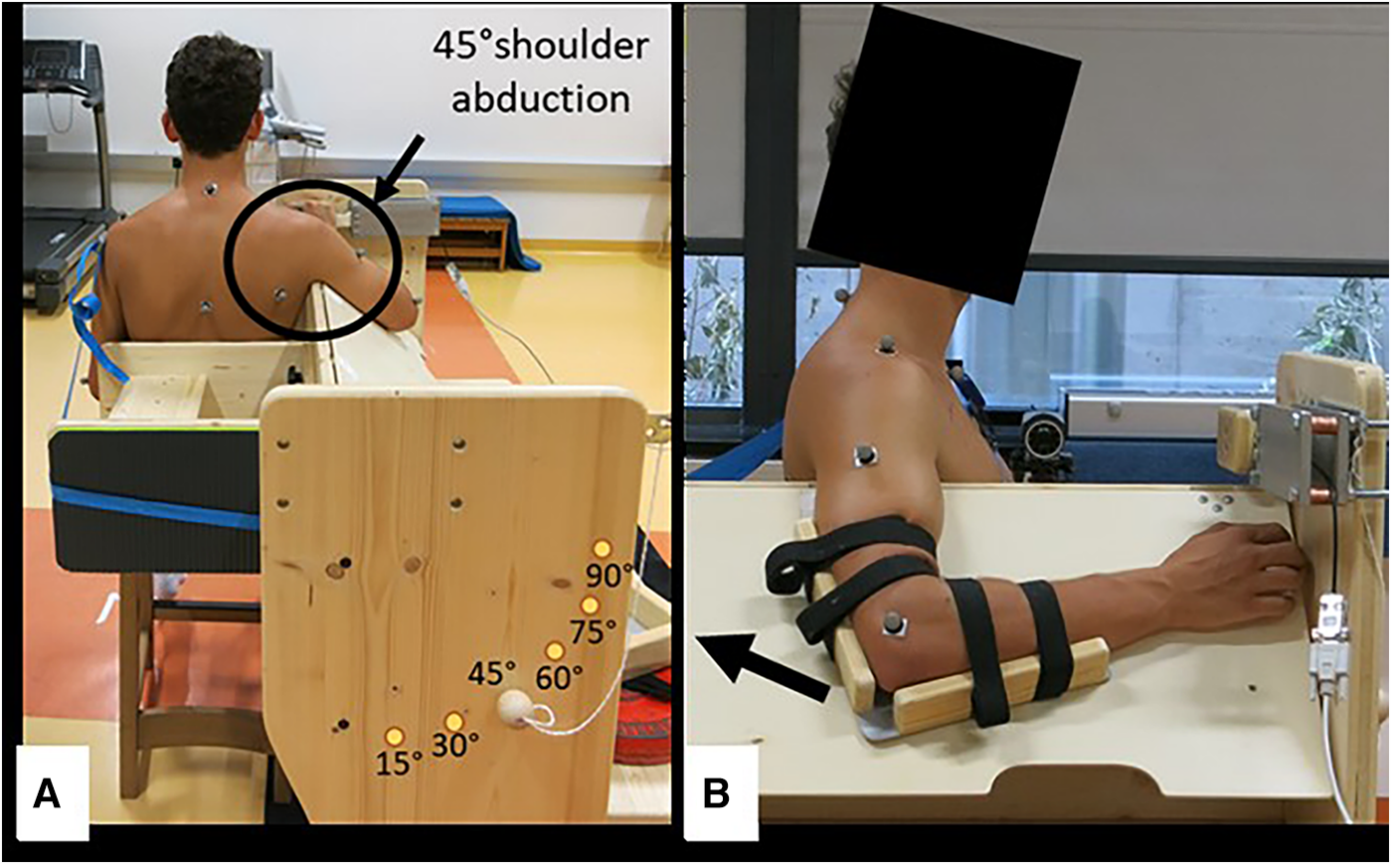
Schematic representation of the starting position of the participant during the shoulder extension ROM test. (**A**) Rear perspective showing the shoulder abduction angle; and (**B**) neutral starting position for the ROM test. The arrow shows the direction of motion of the elbow during the test.

A three-dimensional (3D)-motion capture system (Qualisys, Gothenburg, Sweden) was used to test the shoulder extension ROM, while the participant was sitting next to the custom-made device (Figure 1A). According to the Qualisys “CAST upper body marker set” and additional marker of the “CGM upper body marker set,” 16 reflective markers (1 cm diameter) were positioned on each participant's arms and trunk. To keep a 90° elbow angle stable throughout the shoulder extension ROM test, the participant´s arm was strapped to a custom-made fixation (Figure 1B). Moreover, to avoid any accessory movements, the participant's trunk was fixed with a strap to the backrest of the custom-made device. The test was performed with a 45° shoulder abduction angle and, consequently, a board was fixed at this angle to guide the participant’s movement. The starting position was a neutral shoulder joint position, and the participant's task was to move the fixed elbow along the board as far behind the body as possible, while the shoulder was not allowed to be pulled to the neck. The movement was performed at a slow speed and was done three times, with 15 s breaks in between. The recorded markers of the upper arm and the torso were mapped in a consisting model. Visual 3D Professional x64 (C-Motion Inc., Germantown, Virginia, USA) was used to extract the angles of the shoulder joint. The joint angles in all three planes of motion were calculated as the relations of the torso and the upper arm positions to each other. The attempt with the highest shoulder extension ROM was considered for further analysis.

#### 2.3.2 Seven-week static stretching and foam rolling intervention

The intervention was performed for a period of 7 weeks on the dominant leg (e.g., leg used if one would have to kick a ball). A single training session consisted of three exercises, and the participants were asked to participate in three sessions per week (recommendation: Monday, Wednesday, and Friday). Every exercise had to be performed for 5 min, which led to a total training duration of 15 min per session. Using the same stretching protocol as in Konrad et al. (21), Exercise 1 involved a plantar foot sole stretch, wherein the participant stood facing a wall. The toes and ball of the foot were placed against the wall, with the heel remaining on the floor. To create a stretching sensation, the participant pushed the ball of the foot toward the floor, maintaining the position with the highest tolerable stretching intensity. If the stretching intensity was insufficient in a standing position, the participant could either sit down or cross their calf over the opposite thigh. In this seated position, they would induce a plantar facia stretch by pulling their toes and the ball of the foot dorsally until a significant stretching intensity was felt [refer to Figure 2A in Konrad et al. (21)]. Exercise 2 involved a rolling activity using a custom-made wooden cylinder (diameter = 5.5 cm, length = 15 cm). The participant rolled the cylinder continuously along the plantar foot sole, moving linearly between the ball of the foot and the heel at a slow speed (approximately 2 s back and forth). The participant applied as much pressure as they could tolerate [see Figure 2B in Konrad et al. (21)]. Exercise 3 was a similar rolling exercise but utilized a foam roller ball (Ball 08, Blackroll, Bottighofen, CH). The participant was instructed to roll the foam roller ball in small circles constantly and slowly, covering the sole of the foot, between the ball and the heel [see Figure 2C in Konrad et al. (21)]. The pressure applied was to be again as high as the participant could tolerate. Exercises 2 and 3 could be performed while sitting on a chair or standing with support, such as touching a wall or a desk. The participants were advised to maintain the same technique throughout the intervention period.

The participants of the control group were not asked to perform any stretch or foam rolling exercises throughout the intervention period.

### 2.4 Statistical analyses

The statistical analysis was performed using SPSS (version 28, SPSS Inc., Chicago, Illinois). Normal distribution of ROM was confirmed by the Shapiro–Wilk test. Thus, a linear mixed model ANOVA [within factor: time (pre vs. post) and between factor: group (intervention vs. control)] was performed. The partial eta square (*η*^2^) was calculated as the effect size, and *η*^2^ greater than 0.01, 0.06, and 0.14 were interpreted as “small,” “medium,” and “large” effect, respectively (26). The comparison between the baseline characteristics of the intervention group and the control group was performed with an unpaired *t*-test. The alpha level was set to 0.05.

## 3 Results

**Figure 2:**
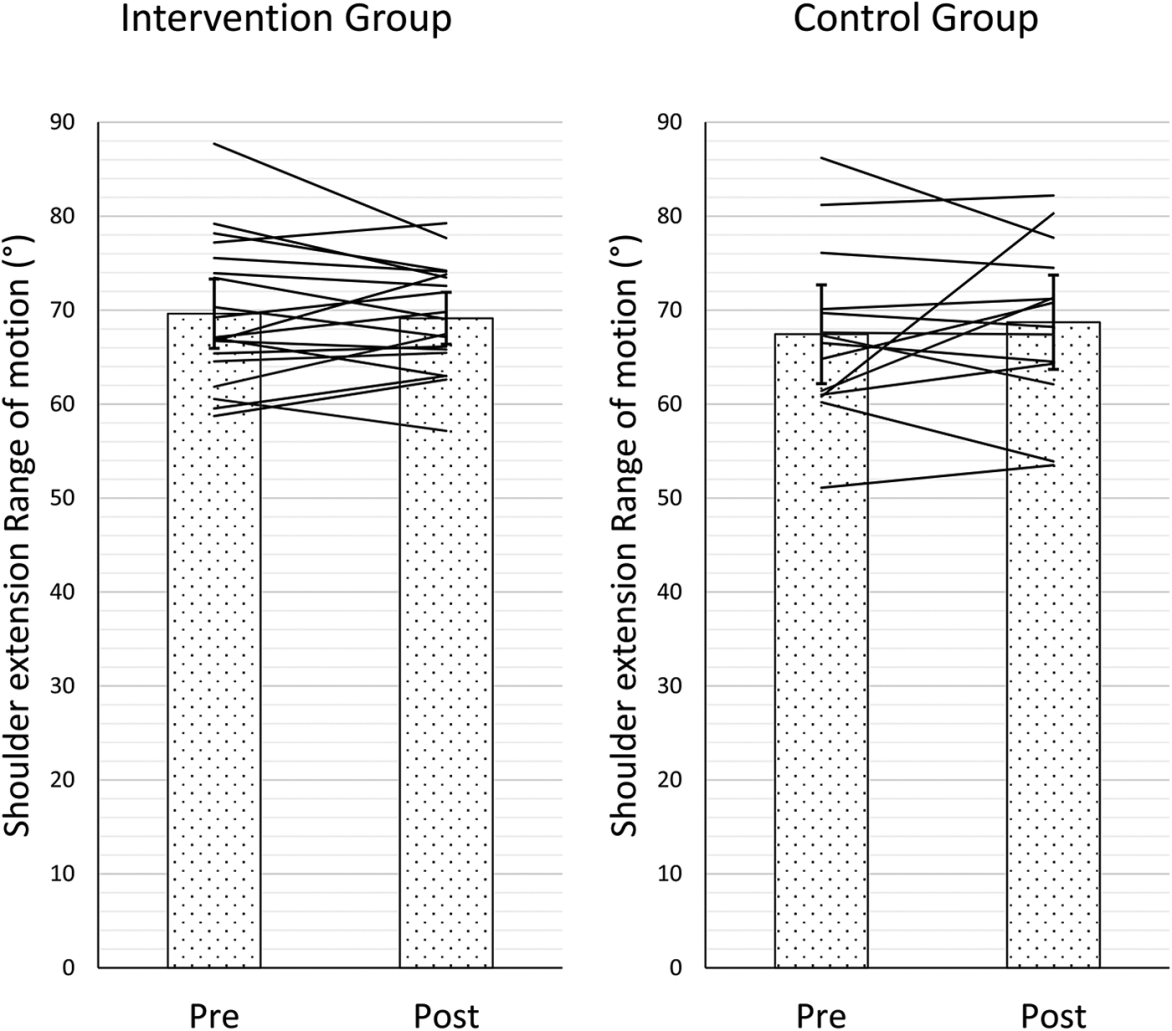
Pre- and postmean ROM of both groups (intervention and control groups).

The ANOVA revealed no significant time (*p* = 0.70, *F*_1,31 _= 0.16; *η*^2^ = 0.01) or time×group effect (*p* = 0.38, *F*_1,31 _= 0.79; *η*^2^ = 0.03) for the shoulder extension ROM. The values of the ROM changed during the observation period in the intervention and control groups from 69.62 ± 7.66°, 67.43 ± 9.11° to 69.13 ± 5.72°, 68.71 ± 8.69°, respectively. The individual values are represented in Figure 2.

## 4 Discussion

The purpose of this study was to investigate the effects of a 7-week combined intervention (i.e., static stretching and foam rolling) of the foot sole on shoulder extension ROM. The results revealed no significant change in shoulder extension ROM following the treatment of the foot sole.

A recent meta-analysis on the non-local effects of single passive stretches reported acute changes in ROM in non-stretched homologous as well as heterologous joints (12). Similarly, a scoping review on the contralateral effects of foam rolling reported an acute increase in ROM in non-rolled homologous joints of 5.60% [confidence interval (95%) 3.65%–8.19%] (20). Both reviews concluded that the non-local changes in ROM are likely attributed to either increased global pain tolerance or simply warm-up effects. An original study reported an acute increase in hamstring extensibility following a single foam rolling intervention of the foot sole, which indicates a potential remote effect along the superficial backline (13). It can be concluded that there is evidence that a single stretching or foam rolling exercise can increase ROM of non-stretched/non-rolled body regions acutely, either because of warm-up effects, an increased global pain tolerance, or because of strain transfer along myofascial chains (i.e., remote effects along the fascia).

Recent research on the chronic non-local effects of stretching and foam rolling on ROM has primarily focused on investigating the contralateral homologous effects of these two methods. For both stretching and foam rolling, an increase in ankle dorsiflexion ROM of the contralateral limb following an intervention of the ipsilateral limb for several weeks was reported (16–19). The assessed contralateral effect was attributed to an increase in stretch tolerance rather than changes in muscle stiffness as reported following static stretch (17) and foam rolling training of the target muscles (19). As seen in recent meta-analyses, it is evident, at least for long-term stretch training (but not for foam rolling), that it can locally (i.e., in the stretched muscle) decrease muscle stiffness (27) and increase fascicle length (28). However, if no stretch stimulus is applied to the contralateral limb, such a change in muscle structure as seen in the stretched limb is unlikely and rather neurological adaptations may occur in the contralateral limb.

Besides the chronic contralateral homologous effects of stretching and foam rolling not much is known about non-local heterologous changes in ROM following stretching or foam rolling training. Only one recent study (21) that used the same intervention on the foot sole as applied in our study (15 min/session combined treatment of stretching and foam rolling—3 times/week for 7 weeks) showed no significant remote effect throughout the superficial backline on ankle ROM (21). However, according to the multivariate comparison of the study of Konrad et al. (21), there was a tendency (*p* = 0.08) of an interaction effect with an increase in dorsiflexion ROM. The pre-to-post comparison showed an increase in ROM with a moderate magnitude of change in the intervention group of 2.0° (*p* = 0.05; *d* = 0.5), without any change in the control group (−0.1°; *p* = 0.62; *d* = 0.1). A potential increase in ROM in the intervention group was underlined by the 95% confidence intervals of −0.03° to 4.0°. Although in the study of Konrad et al. (21) an *a priori* sample size calculation was performed, and hence, an appropriate sample was recruited (*n* = 20 for the intervention group) a rather low *post hoc* power (0.56) was shown in ankle ROM. This indicated that a potential, significant difference might have been overlooked owing to the small sample size. While reviews on the acute contralateral effects of stretching (12) and foam rolling (20) reported increases in contralateral ROM attributed to increases in stretch tolerance, the evidence to date [i.e., study of Konrad et al. (21) as well as the findings in our current study] indicates that chronic (training-related) ROM increases with non-treated heterologous muscles or non-local areas (except for homologous contralateral effects) seem unlikely. Hence, if stretch tolerance is the primary mechanism for acute contralateral ROM improvements, then the effect must be transitory and not applicable to chronic stretch and foam rolling training.

The underlying mechanisms are associated with the cross-education “spillover of neural drive” (callosal access) hypothesis. With this hypothesis, control systems’ adaptations in the cortical, subcortical, and spinal levels related to the trained limb may be accessed by contralateral muscles (29). With the homunculi motoneuron organization, homologous muscles may be situated closer to each other than heterologous motoneurons, permitting better access for this “spillover effect.” With the cross-activation hypothesis, homologous motor network activation results in bilateral activation and adaptations that augment subsequent performance (30). Hence, learning to increase stretch (pain) tolerance in one muscle group would be bilaterally transferred preferentially to the contralateral homologous muscle group than to the heterologous muscle groups. Again, potential non-local heterologous changes of muscle structure such as changes in muscle stiffness are unlikely, since no direct stimulus on the target tissue was applied during the training intervention. This was confirmed by a study by Nakamura et al. (17) who reported an increase in ROM in the contralateral homologous without changes in muscle stiffness. Nevertheless, future studies should take into account both potential mechanisms (mechanical and neurological) if non-local heterologous changes in ROM might be expected owing to a certain stimulus (e.g., stretching, foam rolling, strength training). Such a change in ROM as a result of these interventions could be highly significant for immobilized regions, such as those confined by casts or wheelchairs.

Hence, if the objective is to enhance the ROM of a particular joint, it is advisable to focus on stretching and/or foam rolling of the associated muscle. However, to increase the ROM in the long term, strategies other than stretching or foam rolling were reported to increase ROM to a similar extent. Alizadeh et al. (31), for example, showed in their meta-analysis that frequent resistance training performed within the full ROM can chronically increase joint ROM. Besides the increase in ROM, resistance training certainly has other beneficial effects such as increasing muscle strength and muscle mass, reducing back pain, and enhancing cardiovascular health (32).

This study had some limitations. First, the investigators were not blinded to the group allocation. Second, we only tested active healthy individuals, and hence, we cannot generalize our results to other peer groups. Future studies will have to explore if such treatments lead to different results in a variety of populations (e.g., elderly, sedentary). Third, the only parameter assessed was ROM, and hence, potential mechanisms such as neurological changes (i.e., pain perception) were not explored within this study. However, since there were no changes in non-local ROM, we are confident that no changes in pain perception occurred.

## 5 Conclusion

This study was the first to investigate the effects of a 7-week combined intervention (i.e., static stretching and foam rolling) of the foot sole on the non-local heterologous shoulder extension ROM. The combined static stretch and foam rolling training of the foot sole, performed 3 × 15 min a week, did not induce changes in shoulder extension ROM. Consequently, if the goal is to increase the ROM of a specific joint, a treatment of the associated muscle with either stretching, foam rolling, or strength training is recommended.

## Data Availability

The original contributions presented in the study are included in the article/Supplementary Material, further inquiries can be directed to the corresponding author.
